# Supplementary material for: Anticipation of temporally structured events in the brain
Source: eLife. 2021 Apr 22;10:e64972. doi: 10.7554/eLife.64972 (PMC8169103; doi:10.7554/eLife.64972)
Supplement: Transparent reporting form [file elife-64972-transrepform.docx]

***eLife’s* transparent reporting form**

We encourage authors to provide detailed information *within their submission* to facilitate the interpretation and replication of experiments. Authors can upload supporting documentation to indicate the use of appropriate reporting guidelines for health-related research (see [EQUATOR Network](http://www.equator-network.org/%20)), life science research (see the [BioSharing Information Resource](https://biosharing.org/)), or the [ARRIVE guidelines](http://www.plosbiology.org/article/info:doi/10.1371/journal.pbio.1000412) for reporting work involving animal research. Where applicable, authors should refer to any relevant reporting standards documents in this form.

If you have any questions, please consult our Journal Policies and/or contact us: [editorial@elifesciences.org](mailto:editorial@elifesciences.org).

**Sample-size estimation**

- You should state whether an appropriate sample size was computed when the study was being designed
- You should state the statistical method of sample size computation and any required assumptions
- If no explicit power analysis was used, you should describe how you decided what sample (replicate) size (number) to use

Please outline where this information can be found within the submission (e.g., sections or figure legends), or explain why this information doesn’t apply to your submission:

We used a publicly available dataset – i.e., the sample size was already determined. We expect that the sample size (n=30) is sufficient because it is similar to, or exceeds, the sample size in previous studies that have successfully applied similar techniques in naturalistic datasets (Antony et al., 2020, n=20; Baldassano et al., 2018, n=31; Baldassano et al., 2017, n=18).

**Replicates**

- You should report how often each experiment was performed
- You should include a definition of biological versus technical replication
- The data obtained should be provided and sufficient information should be provided to indicate the number of independent biological and/or technical replicates
- If you encountered any outliers, you should describe how these were handled
- Criteria for exclusion/inclusion of data should be clearly stated
- High-throughput sequence data should be uploaded before submission, with a private link for reviewers provided (these are available from both GEO and ArrayExpress)

Please outline where this information can be found within the submission (e.g., sections or figure legends), or explain why this information doesn’t apply to your submission:

The experiment was performed once and we did not exclude any participants.

**Statistical reporting**

- Statistical analysis methods should be described and justified
- Raw data should be presented in figures whenever informative to do so (typically when N per group is less than 10)
- For each experiment, you should identify the statistical tests used, exact values of N, definitions of center, methods of multiple test correction, and dispersion and precision measures (e.g., mean, median, SD, SEM, confidence intervals; and, for the major substantive results, a measure of effect size (e.g., Pearson's r, Cohen's d)
- Report exact p-values wherever possible alongside the summary statistics and 95% confidence intervals. These should be reported for all key questions and not only when the p-value is less than 0.05.

Please outline where this information can be found within the submission (e.g., sections or figure legends), or explain why this information doesn’t apply to your submission:

Statistical analysis methods are described in detail in sections “Detecting anticipatory signals using an Event Segmentation Model” and “Comparison of event boundaries in brain regions to annotations”. We used permutation tests (and corrected for multiple comparisons by applying a False Discovery Rate correction) for the whole-brain analyses in Figure 2 (and the its related Supplementary Figures) and Figure 5, and used bootstrapping to compute confidence intervals for the optimal lags (page 7). We show group results in figures because our sample size is greater than 10 (n=30). We report all p values for the correlation analyses (pages 4 and 18) for both significant and non-significant correlations, and attached full FDR-corrected p value maps as Source Data.

(For large datasets, or papers with a very large number of statistical tests, you may upload a single table file with tests, Ns, etc., with reference to sections in the manuscript.)

**Group allocation**

- Indicate how samples were allocated into experimental groups (in the case of clinical studies, please specify allocation to treatment method); if randomization was used, please also state if restricted randomization was applied
- Indicate if masking was used during group allocation, data collection and/or data analysis

Please outline where this information can be found within the submission (e.g., sections or figure legends), or explain why this information doesn’t apply to your submission:

The fMRI study was a within-participant design: all participants viewed the same movie clip 6 times each. For the event annotations, all participants provided annotations for the same movie clip (i.e., also a within-participant design). We note this in Methods (section 5).

**Additional data files (“source data”)**

- We encourage you to upload relevant additional data files, such as numerical data that are represented as a graph in a figure, or as a summary table
- Where provided, these should be in the most useful format, and they can be uploaded as “Source data” files linked to a main figure or table
- Include model definition files including the full list of parameters used
- Include code used for data analysis (e.g., R, MatLab)
- Avoid stating that data files are “available upon request”

Please indicate the figures or tables for which source data files have been provided:

Data: <https://openneuro.org/datasets/ds001545/versions/1.1.1> (noted in section “Grand Budapest Hotel dataset”).

Code: <https://github.com/dpmlab/Anticipation-of-temporally-structured-events> (noted in section “Code and resource availability”)

Results: Attached as Source Data, and at <https://neurovault.org/collections/9584/> (noted in section “Code and resource availability”)
